# Repair of contained ventricular rupture with infected intrapericardial thrombus

**DOI:** 10.1093/jscr/rjac301

**Published:** 2022-06-22

**Authors:** Dania Hasan, Zach M DeBoard

**Affiliations:** Elson S. Floyd College of Medicine, Washington State University, Spokane, WA, USA; Elson S. Floyd College of Medicine, Washington State University, Spokane, WA, USA; Division of Cardiac & Thoracic Surgery, Providence Regional Medical Center Everett, Everett, WA, USA; Department of Cardiothoracic Surgery, Waikato Cardiothoracic Surgery Unit, Hamilton, New Zealand

## Abstract

Contained left ventricular rupture, or pseudoaneurysm, is a rare entity resulting from adhesions confining the defect to a localized portion of the pericardial space. Concomitant infection is even more infrequent. We present the first-known case of a patient with an infected intrapericardial thrombus from a left ventricular rupture.

## INTRODUCTION

Left ventricular free wall rupture (LVFWR) secondary to acute myocardial infarction (MI) is thought to occur in <1% of cases [1, 2]. Non-traumatic rupture is attributed to inadequate coronary collateral circulation yielding profound ischemia, with eventual necrosis or scar formation. This entity is infrequent, given current early percutaneous reperfusion techniques. LVFWR is often fatal unless early intervention is pursued. Contained rupture, or pseudoaneurysm, can present with hemodynamic instability and eventual death if not repaired. Even rarer is the direct involvement of bacterial infection with the rupture [3–7]. We report the first case of a 64-year-old patient with an infected intrapericardial thrombus in the setting of contained LVFWR.

## CASE REPORT

A 64-year-old man without prior medical history experienced an inferior-lateral ST-segment elevation MI. He was treated with drug-eluting stent placement to the right coronary artery that was burdened by a large, acute thrombus. On post-procedure day 2, he complained of angina prompting drug-eluting stent placement to the circumflex artery. Subsequent transthoracic echocardiogram was unremarkable and demonstrated an ejection fraction of 55%. He was discharged on aspirin, atorvastatin, metoprolol and ticagrelor.

**Figure 1:**
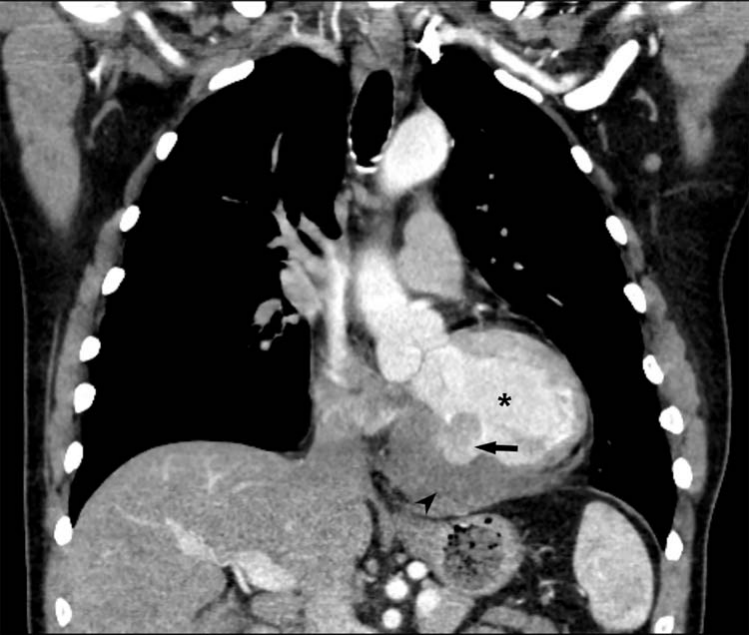
Coronal CT demonstrating left ventricular wall defect and intrapericardial thrombus; asterisk = left ventricular cavity, arrow = left ventricle free wall defect, arrowhead = thrombus.

**Figure 2:**
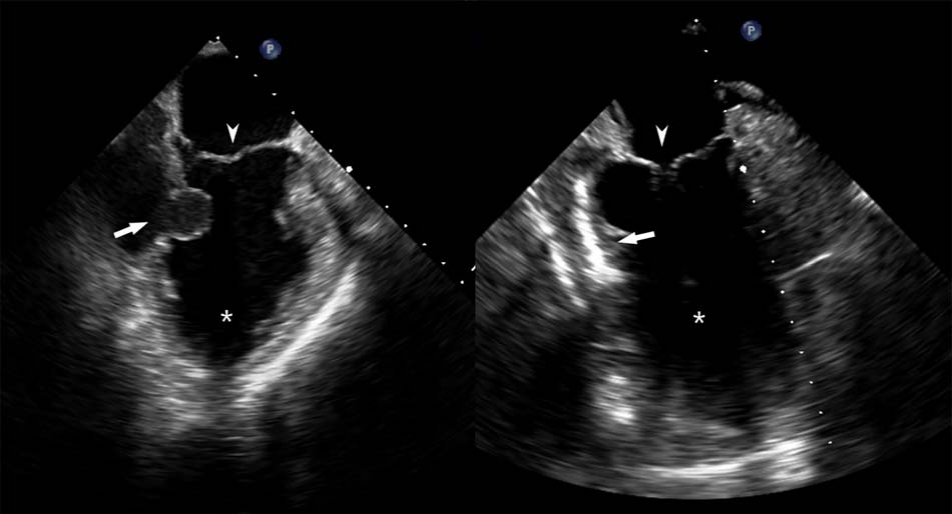
Pre- and post-repair transesophageal echocardiography images; leftward image: asterisk = left ventricular cavity, arrow = ventricular wall defect, arrowhead = mitral valve; rightward image: asterisk = left ventricular cavity, arrow = ventricular wall patch closure, arrowhead = mitral valve.

He returned 5 weeks later complaining of 72 hours of malaise, fevers and chest pressure. He was tachycardic and hypotensive but intermittently responsive to fluid resuscitation. He noted reluctance to present for evaluation, given concerns regarding COVID-19 exposure if he was hospitalized. A computed tomography (CT) of the chest noted a left ventricular irregularity and adjacent thrombus (Fig. 1). Echocardiography revealed an ejection fraction of 35%, a large pericardial effusion and an aneurysmal-appearing inferolateral left ventricular wall with intrapericardial thrombus (Fig. 2).

**Figure 3:**
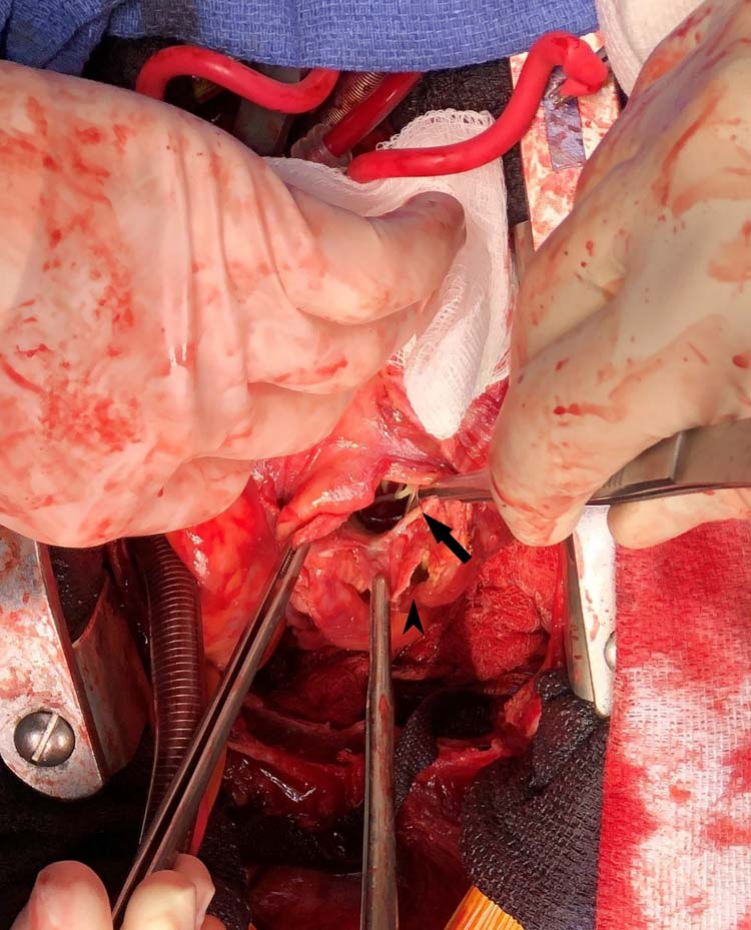
Intraoperative photograph demonstrating left ventricular wall defect; the heart is oriented with the apex retracted cephalad; arrow = left ventricular wall and mitral valve chordae, arrowhead = pseudoaneurysm wall.

Emergent surgical intervention was performed with a median sternotomy, aorto-bicaval cannulation and antegrade cardioplegia. Dense intrapericardial adhesions were lysed, revealing a large pseudoaneurysm sac containing murky, seropurulent fluid. After debridement, a 4-cm defect in the inferolateral wall below the mitral valve was observed (Fig. 3). This was closed with an oversized bovine pericardial patch secured to healthy endocardium with a running polypropylene suture. The pseudoaneurysm edges were re-approximated over the repair in a linear fashion using two layers of suture polypropylene buttressed with felt strips. Post-repair echocardiography demonstrated no residual defect, ventricular geometry distortions or mitral valvular dysfunction (Fig. 2).

Blood and surgical fluid/tissue cultures revealed *Peptostreptococcus asaccharolyticus*, supporting a dental source, treated with 6 weeks of intravenous ertapenem. The patient was discharged on post-operative day 14 and continues to do well 2 years from intervention.

## DISCUSSION

LVFWR occurs in <1% of MI because of early percutaneous revascularization. Patients with LVFWR yield a mortality up to 60% from the resulting cardiac tamponade [1, 2]. In some cases, rupture is contained by adherent pericardium and fibrotic tissues forming a pseudoaneurysm [3–5]. While ventricular pseudoaneurysms can be fatal, they are likely permissive of delayed presentation. Like the current case, reports have demonstrated the inferior or posterolateral ventricular walls as predominant sites of pseudoaneurysm development [1, 2].

Given the profound risk of mortality, early diagnosis and intervention are crucial. In comparison to 50–60% mortality with medical management alone, surgical therapy risks mortality around 10% and lower if without mitral valve intervention [1, 2].

An infected thrombus is unique as limited available reports describe infection of true ventricular aneurysms [3]. Even less common are contained ruptures with coexisting infection [4–8]. A similar, single report describes repair of a LVFWR in the setting of purulent pericarditis rather than an infected intrapericardial thrombus, as in the current case [7]. Both the current case and the aforementioned yielded *Peptostreptococcus*. *Staphylococcus* and *Streptococcus* species predominate as the organisms in the referenced reports [8]. The presence of the anaerobic, gram-positive *Peptostreptococcus* outside of recent dental work or clinically observed dental infection represents a rare, but possible source.

A new phenomenon may be observed with the current case related to the COVID-19 pandemic as the patient delayed pursuit of care out of concern for infectious exposure. Up to 42% of USA adults reported postponing or avoiding medical care during the pandemic and 16.9% of adults would avoid emergency care in scenarios of acute MI out of concern for COVID-19 exposure [9, 10]. This finding may yield increased infrequently encountered conditions such as LVFWR.

## CONFLICT OF INTEREST STATEMENT

None declared.

## FUNDING

None.
